# Oral infections in oral cancer survivors: A mini-review

**DOI:** 10.3389/froh.2022.970074

**Published:** 2022-10-21

**Authors:** Alberto Pispero, Niccolò Lombardi, Maddalena Manfredi, Elena Maria Varoni, Andrea Sardella, Giovanni Lodi

**Affiliations:** ^1^ Dipartimento di Scienze Biomediche, Chirurgiche e Odontoiatriche, Università Degli Studi di Milano, Milan, Italy; ^2^ Dipartimento di Medicina e Chirurgia, Centro di Odontoiatria, Università di Parma, Parma, Italy

**Keywords:** oral cancer, candidiasis, oral infections, cancer survivors, oral tumour

## Abstract

The oral cancer survivors are a group of special individuals whose disease affect anatomical structures with a key role in identity and communication and a fundamental role in basic human functions such as speaking, eating, swallowing and breathing. Thus, quality of life of these individuals can be impaired by the consequences of disease and treatments, in particular surgery and radiotherapy. Among others, infectious conditions of any nature, bacterial, viral, fungal, are a frequent finding among oral cancer survivors. In fact, the peculiar systemic and local conditions of these subjects are known to significantly modify the microbiota, which, besides facilitating opportunistic infections, can affect the cancer microenvironment, as well as alter the effects of the anti-cancer therapies. Similarly, mouth infections can also affect the prognosis of oral cancer survivors. Among the opportunistic infections, fungal are the most common infections affecting these subjects, since neutropenia resulting from cancer, as well as chemotherapy and/or radiotherapy treatments, promote the shift from the carrier state of Candida species, to pathogen state. Treatment of oral candidiasis can be difficult in oral cancer survivors, and good evidence supports clotrimazole as the most effective for prevention, and fluconazole as the one with the best risk-benefit profile. Probiotics, although promising, need better evidence to be considered an effective treatment or preventive measure.

## Introduction

According to the GLOBOCAN 2020 estimates produced by the International Agency for Research on Cancer, in 2020 19.3 million new cancer cases were diagnosed world-wide, a figure that in 20 years' time could reach 28.4 million (1). Thanks to ageing populations, advances in early diagnosis, and treatment effectiveness, the number of cancer survivors is rapidly increasing worldwide. “Cancer survivor” refers to anyone who has ever received a diagnosis of cancer, regardless of where they are in the course of their disease: the estimated 5-year prevalence of all cancers is 50.5 million (2). The Institute of Medicine (nowadays National Academy of Medicine, www.nam.edu) listed the following aims of a correct cancer survivor care: surveillance for recurrence, screening for spreading, or new primary cancers, assessment and management of the consequence of cancer and treatment, health promotion, and coordination between specialists and primary-care providers (3, 4). The oral cavity and pharynx cancer males survivors are about 250,000 in the US only, and they are expected to increase significantly by 2030 (5). Cancer of the mouth and its treatments can affect a number of structures involved in key functions, such as speaking, eating, swallowing, breathing, as well having a central role in identity and communication of the individual. Thus, oncological surgery of the oral and perioral structures, chemotherapy with both traditional and biological drugs, and radiotherapy of the head and neck can significantly affect the quality of life of oral cancer patients (6). In addition, the high proportion of subjects over the age of 65 among them (59%), makes comorbidities highly common, further complicating the follow-up of this special group of cancer survivors (5).

One of the most relevant factors affecting the quality of life of oral cancer survivors is dental and oral health. Infective conditions of the soft and hard tissues of the mouth are common findings among those patients, and they include healthcare-associated infections (7), infectious complication of the surgical site, the most common reason for 30-day all-cause readmissions among patients surgically treated (8), or opportunistic infections which are extremely common during and after cancer treatment. In addition, common infectious conditions of teeth and gums, namely caries and periodontal diseases, have higher incidence, among cancer survivors. The susceptibility to infections of the oral cancer survivors depends on a number of factors including the age of patient, comorbidities, tobacco and alcohol use, oral health conditions at the time of diagnosis, stage and location of cancer, type of surgical intervention, dose and modalities of radiotherapy and consequent hyposalivation, drugs used for chemotherapy. Besides some common infections affecting the oral cancer survivor, this mini-review will address some less debated issues related to the infections in cancer survivors, such as their putative prognostic role and the changes in microbiota of these patients.

## Microbiota modifications in cancer patients

The human microbiome is composed by the genome of the entire microbiota, which is represented by the ecological community of commensal, symbiotic and pathogenic microorganisms residing within and on the human body. Cancer therapies, mainly chemotherapy, immunotherapy and radiotherapy may affect the composition of microbiota, which can mediate both the therapeutic response and toxicity, also predisposing patients to infective complications. Qualitative and quantitative variations in bacterial communities as well as changes in the host environment can transform fungal commensals into opportunistic pathogens in the upper and lower gastrointestinal tract (9). Pioneering studies have shown that Streptococcus oralis has a mutualistic relationship with *C. albicans: C. Albicans* enables streptococcal biofilm growth at mucosal sites, while S. oralis facilitate invasion of the oral and esophageal mucosa by *C. albicans* (9).

In the gut, chemotherapy has been reported to produce severe dysbiosis, which may be further worsened by the concomitant use of antibiotics (10); the latter associated with a decrease the clinical activity of cancer immunotherapy (11). Several gut bacterial taxa appeared protective against the cancer immunotherapy's toxic effects and Bacteroidetes appeared abundant in patients resistant to ipilimumab-induced colitis, and Bifidobacterium can abrogate pathology in a mouse model of immunotherapy-induced colitis (10). Other taxa were, contextually, associated with both immunotherapy success and toxicity as Firmicutes case in immunotherapy and, in preclinical models, the gut dysbiosis associated to oxaliplatin (10). However, the mechanisms through which the gut microbiota influences response to cancer therapies remain not entirely understood (10, 12). About the role of microbiome in immunotherapy, literature supports the interaction of microbial products and components with antigen-presenting cells and innate effectors, which can enhance the adaptive immune response and the induction of cytokine production, besides local or distant effects of microbial metabolites (10). Most of studies focuses on the adaptive immunity induced by the gut microbiota during immunotherapy, suggesting that microorganisms may promote antitumor CD8 + T cell responses during treatment (12). The influence of gut microbiota on TH1 immune response and its modulation of TH17 cells have also been proposed as mechanisms, which may regulate the tumor microenvironment (12). Microbiota can also influence responses to a range of chemotherapy regimen; beneficial responses to cyclophosphamide were associated with increased intestinal permeability, producing bacterial translocation that can result in the maturation of TH17 cells (10).

Radiotherapy is also responsible of a proinflammatory dysbiosis, impairing intestinal mucosa and related functions (13). In preclinical models, radiotherapy changed the composition of gut microbiota, reducing the abundance of Firmicutes and increasing that of Proteobacteria, favoring the susceptibility to radiation-induced colitis (10). No studies directly investigated the impact of gut microbiota on radiotherapy efficacy and little is still known about how it can regulate the response to this cancer therapy (14, 15). However, some studies support that gut microbiota influence normal tissue radiosensitivity. In mice, the disruption of the circadian rhythm led to reduction of gut microbe species, which is associated with increases mouse sensitivity to gamma-ray irradiation (14). This suggests that alterations in gut microbiota may affect the response to radiotherapy modulating radio-sensitivity of the tissue (14). Consistently, gut microbiota also influences the intestinal barriers and modulates the inflammatory responses, which have impact on the sensitivity or resistance of tumors to radiotherapy. On these bases, it has been speculated that gut microbiota may influence the radiotherapy efficacy, although the role of gut microbiota in radio-sensitivity remains a new concept and the underlying mechanisms are still obscure. Much more research is needed on this topic (15).

Oral microbiome has been also investigated, even if literature is still scanty. In 2018, a systematic review (16) evaluated the specific effect of systemic chemotherapy on the microbiota of the oral cavity: 17 studies were included, 5 were on pediatric patients, 12 were on adult patients. They overall reported, during chemotherapy, a higher proportion of gram-negative bacteria of the Enterobacteriaceae family and gram-positive Streptococcus; these variations could predispose the patient to the occurrence of systemic (septicemia or localized infections) and local (acute oral infections, oral mucositis) complications (16). The disruption of the balance between bacterial load and the immune status which is compromised allows certain bacteria and Candida species to multiply and overwhelm other resident microorganisms. Head and neck radiotherapy for oral cancer causes severe alteration in oral microbiota and, after radiotherapy, the patient may acquire drug-resistant opportunistic infections, which may cause systemic complications and high morbidity (17). *Candida albicans* and *Klebsiella species* and *Pediococcus species* are, in particular, the most important pathogens isolated in post-radiotherapy cancer patients (17).

To corroborate these findings, a further clinical study showed that the combined chemotherapy-radiotherapy treatment protocols, often used in case of oral and oropharyngeal cancers at advanced stages, altered the oral microbiome and metabolomic profiles for 24-month post-treatment. Nitric oxide (NO−) homeostasis is crucial to mammalian physiology: as a free radical signaling molecule, NO− regulates cellular processes such as angiogenesis, smooth muscle tone, immune response, apoptosis, and synaptic communication (18). The recently described enterosalivary nitrate–nitrite–nitric oxide pathway has been shown to provide bioactive NO− from dietary nitrate source. This pathway is dependent upon oral nitrate-reducing bacteria, since humans lack this enzyme activity (18). The majority of downregulated metabolites, after chemotherapy-radiotherapy regimens, were nitric oxide-related precursor, modulator, and/or catalyst such as aspartic acid, phenylalanine, L-ornithine, L-proline, xanthine, tyrosine, and glycine (18). The salivary metabolites reflected the oral microbiome communities and supported the hypothesis of the loss of NO- bioavailability in oro-pharyngeal cancer patients post-chemotherapy-radiotherapy, due to the reduction of oral nitrate-reducing bacteria (18). chemotherapy-radiotherapy, indeed, resulted in oral dysbiosis associated with the specific depletion of genera regulating the enterosalivary nitrate–nitrite–nitric oxide pathway (18).

## Opportunistic fungal infections

Cancer patients are at risk in developing opportunistic fungal infections and in particular oral candidiasis (OC) which can involve only the oral cavity or, more often, extend towards the oropharynx and esophagus or result in systemic infection. Neutropenia resulting from cancer as well as chemotherapy and/or radiotherapy treatments will promote the shift from the carrier state of Candida species (*Candida spp*), to pathogen state, leading to clinical debilitating infections (19).

The prevalence of OC in these patients may vary from different studies in literature, depending on populations studied and diagnostic criteria adopted (20). Overall, oropharyngeal candidiasis is reported to be present in 5%–60% of patients affected by solid tumors and in 20%–80% of patients underwent autologous bone marrow transplantation (21).

Diagnosis of OC is mainly based on clinical sign and symptoms in association with medical history of the patient: however, in immunocompromised patients, where fungal infections tend to recur and become chronic, the microbiological evaluation of the *Candida spp* involved, as well as their susceptibility to antifungal treatment, may be of help in the management of these infections, avoiding the emergence of resistant strains (22).

**Figure 1:**
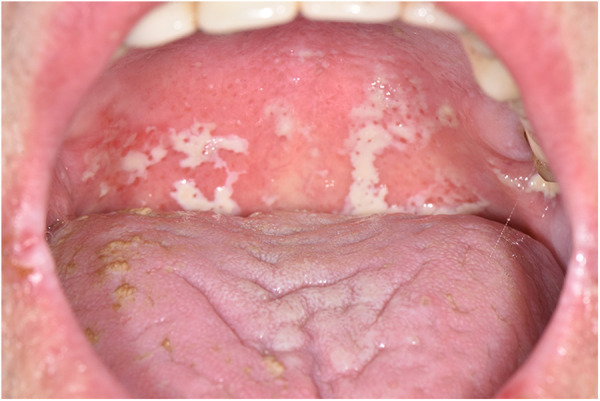
Oral pseudomembranous candidiasis of the soft palate in a cancer patient.

Three main clinical forms of OC have been described in cancer patients: pseudomembranous candidiasis, erythematous candidiasis and angular cheilitis. The most typical and easy to recognize form is the pseudomembranous (also known as oral thrush), characterized by the presence of whitish pseudomembranes that can be removed by scraping and leading to an erythematous base (Figure 1). Lesions tend to spread on all the mucous membranes of the oral cavity including tongue, cheeks, lips, palate and pharyngeal tissues. Often, in patients undergoing chemotherapy or radiotherapy of the head and neck region, this form of OC can also be observed as superinfection of oral mucositis. Erythematous candidiasis may present as acute or chronic form: it is generally associated with broad spectrum antibiotics or corticosteroids, commonly used in cancer patients. Dorsum of the tongue is the most common localization of this infection: it appears as dry, red, and shiny; palate is often simultaneously involved (kissing lesions) (20, 23).

Angular cheilitis is classified as a Candida-associated lesion: the presence of yeast is not the unique etiological agent and bacteria, mainly *Staphylococcus aureus*, are implicated in its etiology. Clinically it appears as chronic erythematous inflammatory lesion of the labial commissures (both unilaterally or bilaterally), with painful fissures that tend to bleed with time.

Symptoms associated with fungal infections can be more or less pronounced, but cancer patients affected by chronic OC complain of burning, dysphagia and difficulty in feeding, with the need to start appropriate antifungal treatment and sometimes to suspend ongoing drug therapies.

The therapy of choice for superficial oral candidiasis is topical, due to a lower risk of side effect and drug interactions, while systemic therapy is generally reserved for recurrent infections or in immunocompromised patients with already extensive infections (19, 24). Polyenes (nystatin and amphotericin B) and some topical forms of azoles (clotrimazole, miconazole) are commonly used for OC: prolonged contact of drugs with the oral mucosa is recommended, repeated several times during the day. It should be noted that not all the formulations of the different antifungal agents are always available: for example, topical formulations of amphotericin B are not available in several countries and its use is reserved as systemic treatment in hospitalized patients with severe fungal infections (Table 1).

**Table 1:** Typical antifungal agents used for the treatment of oral candidiasis in cancer patients.

Antifungal agents	Form	Dosage	Advantages/Disadvantages
POLYENS			
Amphotericin B	Lozenges 10 mg^a^	Dissolve 1 lozenge in the mouth 3–4 times a day after meals	Scarse drug interactions, scarse strain resistance/short duration contact time, highly sucrose sweetened
	Suspension^a^100 mg/ml	Rinse the oral cavity 4–5 times a day after meals	Scarse drug interactions, scarse strain resistance/short duration contact time, highly sucrose sweetened
Nystatin	Pastilles^a^ (200.000 U each)	Dissolve 1 pastille in the mouth 4 times a day after meals for 7–14 days	Scarse drug interactions, scarse strain resistance/short duration contact time, highly sucrose sweetened
	Suspension (100.000 U/ml)	Rinse the oral cavity with 4–6 ml 4–5 times a day after meals for 21 days	Scarse drug interactions, scarse strain resistance/short duration contact time, highly sucrose sweetened
AZOLES			
Myconazole	Gel 2%, cream 2%	Apply directly on the interested area	Low risk of fungal resistance, once daily application/possible drug interactions
	Mucoadhesive tablets, 50 mg	Apply 1 tablet a day on the canine fossa for 7–14 days	Low risk of fungal resistance, once daily application/possible drug interactions
Fluconazole	Suspension 50 mg/5 ml	Rinse the oral cavity with 4–6 ml 4–5 times a day after meals for 21 days	Risk of fungal resistance, possible drug interactions
	Capsules 100–200 mg	1–2 tablets daily for 7–14 days	Indicated for mild to severe diseases/Risk of fungal resistance, possible drug interactions
Clotrimazole	Troches 10 mg	Dissolve 1 troche in the oral cavity 5 times daily	Risk of fungal resistance, possible drug interactions
	Cream 1%	Apply to affected area 2–3 times daily for 3–4 weeks	Risk of fungal resistance, possible drug interactions
Itraconazole	Capsules 100 mg	1–2 capsules daily for 7–14 days	Indicated in fluconazole-resistant diseases, possible drug interactions
Ketaconazole	Cream 2%	Apply to affected area 2–3 times daily for 3–4 weeks	Possible skin irritations and headache
	Oral tablets 200–400 mg	4 tablets daily for 14 days	Indicated in systemic fungal infections; can cause severe hepatotoxicity, potential teratogenicity, possible drug interactions

^a^ Not available in all countries.

Adapted from lombardi et al 2020 (25).

Due to the frequency of fungal infections in cancer patients, the need to evaluate the efficacy of treatments that can prevent the onset of OC in these patients has emerged in the literature. In particular, a recent systematic review and network meta-analysis on 20 RCTs (26), reported that clotrimazole, compared with placebo, was the most effective antifungal agent in preventing OC, while fluconazole has the most risk-benefit profile. Unfortunately, there were no RCTs comparing clotrimazole with other antifungal agents.

Finally, the use of some probiotics species in preventing and treating oral and oropharyngeal candidiasis in different patient populations has been investigated in several studies (27–29). In particular, recently, a RCT investigated the effect of probiotic bacteria on oral *Candida spp*. counts in a group of patients who underwent head- and neck- radiotherapy, suggesting that probiotics were effective, alone or in combination with conventional therapies, in reducing *Candida spp* (30). However, although promising, many of these studies were not specifically targeted at evaluating the effect of specific probiotics on a particular host immunodeficiency status. Furthermore, further researches are needed to identify more clearly the inhibitory effect of probiotics on *Candida spp*. in the oral cavity (31, 32).

## Other opportunistic infections

During oral cancer therapy, the neutrophil reduction can put patients at risk for bacterial infections, particularly odontogenic infections. Moreover, the mucositis resulting from chemotherapy represents a big gateway for bacteria into the bloodstream. The inflammatory response is altered, and in the case of infections, the clinical manifestations can be highly variable (33). Besides, the chances of maintaining proper oral hygiene are compromised and depend on numerous factors. The invasiveness of the surgery often limits the opening of the oral cavity and the possibility of accessing the posterior areas. In addition, the onset of mucositis can make oral hygiene maneuvers very painful. These variables, in addition to the reduction of salivary flow, increase the risk of caries and endodontic lesions and expose patients to the onset of periodontal and peri-implant infections. Progression of periapical infections that are untreated or unresponsive to treatment may lead to osteomyelitis of the jaws, resulting in swelling, pain, suppuration, sinus tract formation, bone sequestration, and a radiographically characteristic “moth-eaten” appearance. There are numerous bacteria which constitute normal oral flora, but which may become pathogenic with immune suppression and can cause sepsis: *Viridans Strep*, *Prevotellae*, *Fusobacterium*, *Actinobacillus actinomycetemcomitans*, and *Actinomyces species* may cause oral mucosal infections (34).

The impaired T-cell activity also exposes to viral infections. The oral viral infections include Herpes Simplex virus (HSV), Varicella Zoster virus (VZV), Epstein–Barr virus (EBV), and Cytomegalovirus are often complications of oral cancer treatments. The most common infection is Herpes Simplex. It is frequent in cases where the patient has also undergone chemotherapy in addition to radiation therapy. In particular, chemotherapy seems to be the main cause of the appearance of herpetic lesions. Depending on the patient's level of immunosuppression, exuberant clinical manifestations may occur, such as to confuse these lesions with mucositis or aphthous ulcers (35). Herpes Zoster can induce chickenpox when first infected, and then remain dormant in the neuron of a dorsal root ganglion or a cranial nerve. Later in life, or under a state of compromised immunity, the virus can re-emerge and trigger a unilateral, painful, vesicular rash along the distribution of a dermatome. Oral cancer is associated with Herpes Zoster and in particular, radiotherapy appears to increase the incidence of Herpes Zoster infection in oral cavity (36). Infection by EBV is known to cause infectious mononucleosis and is associated with many human lymphoid and epithelial cancers. The viral prevalent rates varied greatly, ranging from 15% to 77% (37) but the etiologic and tumorigenic roles of the virus in oral cancer remain unclear. Cytomegalovirus infection symptoms are generally evident in immunocompromised patients. Intra-oral lesions appear as nonspecific painful ulcers, usually present for weeks or months, on any mucosal surface. They are often mistaken for, or co-infected with, other viral or fungal infections (38).

## Infections as prognostic factors in oral squamous cell carcinoma

### Viral infections

At least 3% of oral cancer and 30%–60% of oropharyngeal carcinoma cases are possibly caused by HPV infection even though medical literature is still controversial (39, 40). A recent meta-analysis (41) found that HPV-positive status is an adverse prognostic factor for oral squamous cell carcinoma (OSCC), in contrast to the literature demonstrating that HPV-positive oropharyngeal squamous cell carcinomas patients have favourable treatment and survival outcomes. In particular, the result of this meta-analysis showed that the overall survival decreased in HPV-positive OSCC patients compared with HPV-negative. Furthermore, there was also significant decrease of distant control, i.e., metastases, for the patients with HPV-positive OSCC. Exploring the reasons of the difference, the Authors suggested that the prevailing variant of HPV infection in OSCC could be different from those found in other areas of head and neck HPV-positive. Also, the inactivation of HPV genetic expression p16 *via* environment factors (tobacco and alcohol) could also contribute to the different prognosis.

Of interest, could be the possible role of HPV vaccination strategies in patients suffering from head and neck squamous cell carcinoma (HNSCC). While a recent review and meta-analysis demonstrates that adjuvant HPV vaccination is associated with a reduced risk of cervical cancer and cervical intraepithelial neoplasia recurrence, the role of HPV vaccination on primary lesions and recurrences of OSCC remain unknown (42). Nevertheless, this strategy is considered promising since the FDA have recently included prevention of HNSCC among the indications for the 9-valent vaccine (https://clinicaltrials.gov/ct2/show/study/NCT04199689), and several studies are present in literature (43–45). To note, in a recent letter to the Editor, Yilong Hao and Colleagues reported, after the HPV vaccination, the appearance of a symptomatic form of oral lichen planus, a potentially malignant disorder (46).

The Epstein-Barr virus (EBV) was the first human virus associated to oncogenic potential. EBV infects approximately 90% of the world's adult population asymptomatically, and although EBV's role in oral carcinogenesis has not been established yet, its etiological role has been demonstrated in hairy leukoplakia, nasopharyngeal cancer, Burkitt's lymphoma, Hodgkin's disease, and B-cell lymphoma. In a paper aimed to collect data on the prevalence of EBV DNA in patients with OSCC, oral lichen planus, and oral leukoplakia in an eastern Hungarian population, the Authors (47) found that Epstein–Barr virus-positive and EBV-negative OSCC patients did not statistically differ in patient characteristics and exposure to risk factors (smoking and alcohol consumption). Furthermore, the presence of EBV in the tissues of the oral diseases and in OSCC did not increase, respectively, the risk of poor outcome or not influenced the survival (48).

Regarding the Human Immunodeficiency Virus-1 (HIV-1), the introduction of the antiretroviral combined therapy reduced the incidence of AIDS-associated cancers, in particular, Kaposi sarcoma and non-Hodgkin lymphoma. On the contrary, some others cancers, the so-called non-AIDS-defining cancers, have increased significantly (49). Among these, head and neck squamous cell cancers. A recent paper (50) explored the prognostic significance of the HIV infection in patient with head and neck cancer. The Authors, considering age at initial diagnosis, localization, and stage, found a significant difference in both overall survive and disease-free survival rates between patient living with HIV infection and HIV-negative well-matched patients.

### Fungal infections

It is well known that chronic hyperplastic candidiasis is of particular significance due to the potential of malignant transformation that could be, in untreated patients, as high as 10% of the cases (51). Furthermore, several studies showed that Candida species are prevalent in oral squamous cell cancer patients as expected. Regarding the potential effects on oral cancer prognosis of fungal infection, recently, in a population of 100 patients suffering from OSCC the Authors did not observe a statistically significant effect of the yeast on the mortality rate (52).

However, the role of *Candida spp*. in the process of oncogenesis it has been studied and various pathogenic mechanisms involved in epithelial transformation have been investigated (53). Recently, candidalysin, a cytolytic toxin peptide exclusively secreted by pathogenical hyphal forms of *C. albicans*, it has been reported to be essential in epithelial damage and host recognition of candidiasis. This toxin is encoded by ECE1 gene, associated with fungal filamentation and host cell adhesion. Candidalysin damages the epithelial cells, inducing innate immune host response and promoting the expression of cytokines that can contribute to carcinogenesis (54, 55).

### Bacterial infections

Syphilis is a chronic systemic infectious disease caused by the spirochaetal bacterium Treponema pallidum, that has predominant muco-cutaneous lesions, with or without systemic symptoms after the involvement of internal organs. In a recent paper (56), the authors aimed to verify if syphilis has an influence on prognosis in patients suffering from OSCC. Data were retrieved from the TriNetX network, a database that includes clinical data from many health care organizations from different countries. This study did not show a negative influence of syphilis on the five-year survival rate of patients with OSCC, compared to patients without syphilis.

## Discussion

In order to assure a healthy mouth and a good quality of life, the management of oral cancer survivors requires a multidisciplinary approach and the involvement of specialist and non-specialist health care providers.

Because of a number of local and systemic conditions, subjects who received a diagnosis of mouth cancer, and have been treated for that, can be at high risk of local infections of any nature: bacterial, viral, mycotic, that can affect the wellbeing of these subjects, and complicate the course of the disease.

Thus, any health care provider involved in the management of oral cancer survivors should be aware of the infectious conditions that they can face and of the consequences that they might have on the oral and general health of this fragile subjects. That is clearly expressed by the American Cancer Society Head and Neck Cancer Survivorship Care Guidelines (endorsed by the American Society of Clinical Oncology) (57): besides recommending “to maintain close follow-up with the dental professional”, since “preventive care can help reduce caries and gingival disease”, they state that “primary care clinicians should refer head and neck cancer survivors to a qualified dental professional for treatment and management of complicated oral conditions and infections” (recommendation 3.20) (58).

## References

[1] Sung H Ferlay J Siegel RL Laversanne M Soerjomataram I Jemal A Global cancer statistics 2020: GLOBOCAN estimates of incidence and mortality worldwide for 36 cancers in 185 countries. CA Cancer J Clin. (2021) 71(3):209–49. 10.3322/caac.21660 33538338

[2] Emery J Butow P Lai-Kwon J Nekhlyudov L Rynderman M Jefford M. Management of common clinical problems experienced by survivors of cancer. Lancet. (2022) 399(10334):1537–50. 10.1016/S0140-6736(22)00242-2 35430021

[3] Hewitt M Greenfield S Stovall E. From cancer patient to cancer survivor. From Cancer Patient Cancer Surviv. Washington, DC: The National Academies Press (2005):1–506. 10.17226/11468

[4] National Academy of Medicine. www.nam.edu Accessed on 29 August 2022.

[5] Miller KD Nogueira L Mariotto AB Rowland JH Yabroff KR Alfano CM Cancer treatment and survivorship statistics, 2019. CA Cancer J Clin. (2019) 69(5):363–85. 10.3322/caac.21565 31184787

[6] Lombardi N Varoni E Villa G Salis A Lodi G. Pentoxifylline and tocopherol for prevention of osteoradionecrosis in patients who underwent oral surgery: a clinical audit. Spec Care Dent. (2022) 7(1):37–72. 10.1111/scd.12759 35895902

[7] Sankaran SP Villa A Sonis S. Healthcare-associated infections among patients hospitalized for cancers of the lip, oral cavity and pharynx. Infect Prev Pract. (2021) 3(1):100115. 10.1016/j.infpip.2021.100115 34368735 PMC8336044

[8] Goel AN Raghavan G St John MA Long JL. Risk factors, causes, and costs of hospital readmission after head and neck cancer surgery reconstruction. JAMA Facial Plast Surg. (2019) 21(2):137–45. 10.1001/jamafacial.2018.1197 30418467 PMC6439803

[9] Bertolini M Dongari-Bagtzoglou A. The relationship of Candida albicans with the oral bacterial microbiome in health and disease. Adv Exp Med Biol. (2019) 1197:69–78. 10.1007/978-3-030-28524-1_6 31732935

[10] Helmink BA Khan MAW Hermann A Gopalakrishnan V Wargo JA. The microbiome, cancer, and cancer therapy. Nat Med. (2019) 25(3):377–88. 10.1038/s41591-019-0377-7 30842679

[11] Derosa L Hellmann MD Spaziano M Halpenny D Fidelle M Rizvi H Negative association of antibiotics on clinical activity of immune checkpoint inhibitors in patients with advanced renal cell and non-small-cell lung cancer. Ann Oncol. (2018) 29(6):1437–44. 10.1093/annonc/mdy103 29617710 PMC6354674

[12] Zhou CB Zhou YL Fang JY. Gut microbiota in cancer immune response and immunotherapy. Trends Cancer. (2021) 7(7):647–60. 10.1016/j.trecan.2021.01.010 33674230

[13] Gerassy-Vainberg S Blatt A Danin-Poleg Y Gershovich K Sabo E Nevelsky A Radiation induces proinflammatory dysbiosis: transmission of inflammatory susceptibility by host cytokine induction. Gut. (2018) 67(1):97–107. 10.1136/gutjnl-2017-313789 28438965

[14] Al-Qadami G Van Sebille Y Le H Bowen J. Gut microbiota: implications for radiotherapy response and radiotherapy-induced mucositis. Expert Rev Gastroenterol Hepatol. (2019) 13(5):485–96. 10.1080/17474124.2019.1595586 30907164

[15] Liu J Liu C Yue J. Radiotherapy and the gut microbiome: facts and fiction. Radiat Oncol. (2021) 16(1):9. 10.1186/s13014-020-01735-9 PMC7805150 33436010

[16] Villafuerte KRV Martinez CdJ Dantas FT Carrara HHA dos Reis FJC Palioto DB. The impact of chemotherapeutic treatment on the oral microbiota of patients with cancer: a systematic review. Oral Surg Oral Med Oral Pathol Oral Radiol. (2018) 125(6):552–66. 10.1016/j.oooo.2018.02.008 29566996

[17] Anjali K Arun A Bastian T Parthiban R Selvamani M Adarsh H. Oral microbial profile in oral cancer patients before and after radiation therapy in a cancer care center -A prospective study. J Oral Maxillofac Pathol. (2020) 24(1):117–24. 10.4103/jomfp.JOMFP_213_19 32508459 PMC7269272

[18] Lim Y Tang KD Karpe AV Beale DJ Totsika M Kenny L Chemoradiation therapy changes oral microbiome and metabolomic profiles in patients with oral cavity cancer and oropharyngeal cancer. Head Neck. (2021) 43(5):1521–34. 10.1002/hed.26619 33527579

[19] Lalla RV Latortue MC Hong CH Ariyawardana A D'Amato-Palumbo S Fischer DJ A systematic review of oral fungal infections in patients receiving cancer therapy. Support Care Cancer. (2010) 18(8):985–92. 10.1007/s00520-010-0892-z 20449755 PMC2914797

[20] Bensadoun RJ Patton LL Lalla RV Epstein JB. Oropharyngeal candidiasis in head and neck cancer patients treated with radiation: update 2011. Support Care Cancer. (2011) 19(6):737–44. 10.1007/s00520-011-1154-4 21479787

[21] Davies AN Brailsford SR Beighton D. Oral candidosis in patients with advanced cancer. Oral Oncol. (2006) 42(7):698–702. 10.1016/j.oraloncology.2005.11.010 16527512

[22] Manfredi M Polonelli L Giovati L Alnuaimi A McCullough M. Oral and maxillofacial infections In: Contemporary Oral Medicine. (2019).

[23] Singh GK Capoor MR Nair D Bhowmik KT. Spectrum of fungal infection in head and neck cancer patients on chemoradiotherapy. J Egypt Natl Canc Inst. (2017) 29(1):33–7. 10.1016/j.jnci.2017.01.006 28258917

[24] Pappas PG Kauffman CA Andes DR Clancy CJ Marr KA Ostrosky-Zeichner L Clinical practice guideline for the management of candidiasis: 2016 update by the infectious diseases society of America. Clin Infect Dis. (2015) 62(4):e1–e50. 10.1093/cid/civ933 26679628 PMC4725385

[25] Lombardi A Ouanounou A. Fungal infections in dentistry: clinical presentations, diagnosis, and treatment alternatives. Oral Surg Oral Med Oral Pathol Oral Radiol. (2020) 130(5):533–46. 10.1016/j.oooo.2020.08.011 32907786

[26] Shen Loo Y Yee Wong T Veettil SK Se Wong P Gopinath D Mooi Ching S Antifungal agents in preventing oral candidiasis in clinical oncology: a network meta-analysis. Oral Dis. (2021) 27(7):1631–43. 10.1111/odi.13588 32762108

[27] Ai R Wei J Ma D Jiang L Dan H Zhou Y A meta-analysis of randomized trials assessing the effects of probiotic preparations on oral candidiasis in the elderly. Arch Oral Biol. (2017) 83:187–92. 10.1016/j.archoralbio.2017.04.030 28783552

[28] Mundula T Ricci F Barbetta B Baccini M Amedei A. Effect of probiotics on oral candidiasis: a systematic review and meta-analysis. Nutrients. (2019) 11(10):2449. 10.3390/nu11102449 PMC6836010 31615039

[29] Hu L Zhou M Young A Zhao W Yan Z. In vivo effectiveness and safety of probiotics on prophylaxis and treatment of oral candidiasis: a systematic review and meta-analysis. BMC Oral Health. (2019) 19(1):140. 10.1186/s12903-019-0841-2 31291932 PMC6621984

[30] Doppalapudi R Vundavalli S Prabhat M. Effect of probiotic bacteria on oral Candida in head- and neck-radiotherapy patients: a randomized clinical trial. J Cancer Res Ther. (2020) 16(3):470–7. 10.4103/jcrt.JCRT_334_18 32719253

[31] Archambault LS Dongari-Bagtzoglou A. Probiotics for oral candidiasis: critical appraisal of the evidence and a path forward. Front Oral Heal. (2022) 3:880746. 10.3389/froh.2022.880746 PMC9046664 35495563

[32] Contaldo M Di Stasio D Romano A Fiori F Della Vella F Rupe C Oral candidiasis and novel therapeutic strategies: antifungals, phytotherapy, probiotics, and photodynamic therapy. Curr Drug Deliv. (2022) 19. 10.2174/1567201819666220418104042 35440307

[33] Mosel DD Bauer RL Lynch DP Hwang ST. Oral complications in the treatment of cancer patients. Oral Dis. (2011) 17(6):550–9. 10.1111/j.1601-0825.2011.01788.x 21306481

[34] Hong CHL Napeñas JJ Hodgson BD Stokman MA Mathers-Stauffer V Elting LS A systematic review of dental disease in patients undergoing cancer therapy. Support Care Cancer. (2010) 18(8):1007–21. 10.1007/s00520-010-0873-2 20449756 PMC2914291

[35] Elad S Zadik Y Hewson I Hovan A Correa MEP Logan R A systematic review of viral infections associated with oral involvement in cancer patients: a spotlight on Herpesviridea. Support Care Cancer. (2010) 18(8):993–1006. 10.1007/s00520-010-0900-3 20544224

[36] Kao YS Hsu Y Hsu CY. Radiotherapy increases the incidence of herpes zoster in oral cavity cancer patients - A national population-based cohort study. In Vivo (Brooklyn). (2021) 35(6):3547–53. 10.21873/invivo.12657 PMC8627721 34697193

[37] D’Costa J Saranath D Sanghvi V Mehta AR. Epstein-Barr virus in tobacco-induced oral cancers and oral lesions in patients from India. J Oral Pathol Med. (1998) 27(2):78–82. 10.1111/j.1600-0714.1998.tb02098.x 9526734

[38] Myerson D Hackman RC Nelson JA Ward DC McDougall JK. Widespread presence of histologically occult cytomegalovirus. Hum Pathol. (1984) 15(5):430–9. 10.1016/S0046-8177(84)80076-3 6327494

[39] Wierzbicka M San Giorgi MRM Dikkers FG. Transmission and clearance of human papillomavirus infection in the oral cavity and its role in oropharyngeal carcinoma – A review. Rev Med Virol. (2022) 2022(1–9):e2337. 10.1002/rmv.2337 PMC10078185 35194874

[40] Varoni EM Lombardi N Franchini R D'Amore F Noviello V Cassani B Oral human papillomavirus (HPV) and sexual behaviors in a young cohort of oral cancer survivors. Oral Dis. (2021) 27(4):919–23. 10.1111/odi.13622 32871033

[41] Christianto S Li KY Huang TH Su YX. The prognostic value of human papilloma virus infection in oral cavity squamous cell carcinoma: a meta-analysis. Laryngoscope. (2022) 132(9):1760–70. 10.1002/lary.29996 34953144

[42] Di Donato V Caruso G Bogani G Cavallari EN Palaia G Perniola G HPV vaccination after primary treatment of HPV-related disease across different organ sites: a multidisciplinary comprehensive review and meta-analysis. Vaccines. (2022) 10(2):239,2–16. 10.3390/vaccines10020239 35214697 PMC8879645

[43] Schneider K Grønhøj C Hahn CH von Buchwald C. Therapeutic human papillomavirus vaccines in head and neck cancer: a systematic review of current clinical trials. Vaccine. (2018) 36(45):6594–605. 10.1016/j.vaccine.2018.09.027 30268734

[44] Diana G Corica C. Human papilloma virus vaccine and prevention of head and neck cancer, what is the current evidence? Oral Oncol. (2021) 115:105168. 10.1016/j.oraloncology.2020.105168 33730628

[45] Merck Sharp / Dohme LLC. Efficacy against oral persistent infection, immunogenicity and safety of the 9-valent human papillomavirus vaccine (9vHPV) in Men Aged 20-45 Years (V503-049). https://clinicaltrials.gov/ct2/show/study/NCT04199689 (Accessed on August 29, 2022).

[46] Hao Y Yuan Y Li Y Chen Q. Human papillomavirus vaccination induced oral lichen planus. Oral Dis. (2022). 10.1111/odi.14190 35298066

[47] Kis A Fehér E Gáll T Tar I Boda R Tóth ED Epstein-Barr virus prevalence in oral squamous cell cancer and in potentially malignant oral disorders in an eastern Hungarian population. Eur J Oral Sci. (2009) 117(5):536–40. 10.1111/j.1600-0722.2009.00660.x 19758249

[48] Carpén T Syrjänen S Jouhi L Randen-Brady R Haglund C Mäkitie A Epstein–Barr virus (EBV) and polyomaviruses are detectable in oropharyngeal cancer and EBV may have prognostic imact. Cancer Immunol Immunother. (2020) 69(8):1615–26. 10.1007/s00262-020-02570-3 32314041 PMC7347695

[49] Proulx J Ghaly M Park IW Borgmann K. HIV-1-Mediated acceleration of oncovirus-related non-AIDS-defining cancers. Biomedicines. (2022) 10(4):768. 10.3390/biomedicines10040768 35453518 PMC9024568

[50] Haase K Piwonski I Stromberger C Thieme N Heiland M Beck-Broichsitter B Incidence and survival of HNSCC patients living with HIV compared with HIV-negative HNSCC patients. Eur Arch Oto-Rhino-Laryngology. (2021) 278(10):3941–53. 10.1007/s00405-020-06573-9 PMC8382606 33492419

[51] Zhang W Wu S Wang X Gao Y Yan Z. Malignant transformation and treatment recommendations of chronic hyperplastic candidiasis—a six-year retrospective cohort study. Mycoses. (2021) 64(11):1422–8. 10.1111/myc.13371 34553417

[52] Mäkinen A Nawaz A Mäkitie A Meurman JH. Role of non-albicans Candida and Candida albicans in oral squamous cell cancer patients. J Oral Maxillofac Surg. (2018) 76(12):2564–71. 10.1016/j.joms.2018.06.012 30509395

[53] Di Cosola M Cazzolla AP Charitos IA Ballini A Inchingolo F Santacroce L. Candida albicans and oral carcinogenesis. A brief review. J Fungi. (2021) 7(6):476. 10.3390/jof7060476 PMC8231483 34204731

[54] Ho J Camilli G Griffiths JS Richardson JP Kichik N Naglik JR. Candida albicans and candidalysin in inflammatory disorders and cancer. Immunology. (2021) 162(1):11–6. 10.1111/imm.13255 32880925 PMC7730014

[55] Engku Nasrullah Satiman EAF Ahmad H Ramzi AB Abdul Wahab R Kaderi MA Wan Harun WHA The role of Candida albicans candidalysin ECE1 gene in oral carcinogenesis. J Oral Pathol Med. (2020) 49(9):835–41. 10.1111/jop.13014 32170981

[56] Hertel M Hagedorn L Schmidt-Westhausen AM Dommisch H Heiland M Preissner R Comparison of five-year survival rates among patients with oral squamous cell carcinoma with and without association with syphilis: a retrospective case-control study. BMC Cancer. (2022) 22(1):454. 10.1186/s12885-022-09583-4 35468757 PMC9038517

[57] Cohen EEW LaMonte SJ Erb NL Beckman KL Sadeghi N Hutcheson KA American cancer society head and neck cancer survivorship care guideline. CA Cancer J Clin. (2016) 66(3):203–39. 10.3322/caac.21343 27002678

[58] Nekhlyudov L Lacchetti C Davis NB Garvey TQ Goldstein DP Nunnink JC Head and neck cancer survivorship care guideline: American society of clinical oncology clinical practice guideline endorsement of the American cancer society guideline. J Clin Oncol. (2017) 35(14):1606–21. 10.1200/JCO.2016.71.8478 28240970

